# Impact of seasonal changes in urban green spaces with diverse vegetation structures on college students' physical and mental health

**DOI:** 10.1038/s41598-024-67075-w

**Published:** 2024-07-15

**Authors:** Yifan Duan, Hua Bai, Le yang, Shuhua Li, Qiuchen Zhu

**Affiliations:** 1 College of Architecture, Chang’an University, Xi’an, 710061 Shaanxi China; 2 College of Architecture, Tsinghua University, Beijing, 100084 China

**Keywords:** Healthy landscape, Vegetation types, Season, Perception and experience, Environmental perception, Human behaviour, Quality of life, Fatigue

## Abstract

Based on the perceptions of college student participants in winter and summer, the effects of different vegetation structures within landscapes (single-layer woodland, tree-shrub-grass composite woodlands, tree-grass composite woodland, and single-layer grassland) and concrete squares without plants were investigated, and the skin conductivity level (SCL) and environmental perception recovery score (PRS) associated with landscape types were calculated. The results indicated that seasonal differences in landscape perception significantly affected college student participants' PRS but not their SCL scores, both in winter and summer. Viewing single-layer and tree-shrub-grass composite woodlands in summer, as well as single-layer woodland in winter, enhanced the environmental perception of the college student participants. The restorative effects of the four vegetation types in green spaces were ranked as follows: single-layer woodland, tree-shrub-grass composite woodlands, single-layer grassland, and tree-grass composite woodlands and concrete squares without plants. These findings underscore the importance of considering seasonal variations when choosing plant species for landscaping purposes, with evergreen single-layer woodland being a suitable choice for winter urban landscapes. This provides a scientific basis for assessing landscape perception and preferences in the future.

## Introduction

With societal progress and economic development as well as improvements in infrastructure, people's lifestyles and ideas are changing. The acceleration of the pace of life and the intensification of competition have caused People living in large cities suffer from various pressures due to their studies, work, and daily lives^1^, and Rates of depression, suboptimal health, chronic diseases, and cardiovascular diseases are rising among urban populations^2^. Research indicates that people have a strong preference for natural environments over urbanized, developed areas^3^, and for people living in urban environments, urban green spaces are an important component of well-being but are often in short supply. Furthermore, the availability of urban green spaces is a key factor influencing the well-being and quality of life of city dwellers. Urban green spaces can positively impact well-being and health in numerous ways^4^. Increased activity levels due to contact with nature can provide benefits^5^.

Landscape perception and experience are primarily understood through the study of human–environment interactions and the objective features of the environment. and with the objective environment, Moreover, insights from these studies are integrated with landscape aesthetics theories to examine how people's satisfaction and preferences are shaped by their environmental experiences^6^. Landscape perception and experience have been a continuous focus of research due to their importance in understanding human–environment relationships.^7^. Related studies have explored landscape assessment, planning and design from different perspectives, as this topic has become a vast field with various theoretical directions and approaches^8^. With global urbanisation, understanding public perceptions of and preferences for urban green spaces has become particularly indispensable for promoting human well-being and quality of life through human-centred design^9,10^. Although some of the findings from research on landscape perception and experience have been directly implemented in practice, there are questions about the reliability of the strategies adopted by users, planners or practitioners, which may lead to a mismatch between the public demand for green spaces and the actual design of a city^11^. Landscape perception and experience are strongly dependent on people's visual perception, but various known and unknown factors associated with site perception may influence these strategies. Therefore, comparing the effects of site perception, e.g., site characteristics and time of visit (season), on landscape perception and experience remains challenging.

### Influence of visual experience on environmental perception

People's perception of the outside world is mainly obtained through sensory stimulation, and vision, hearing, smell, and touch are important means for perceiving information^12^. The perception of the environment is particularly important, and people have an innate preference for nature; even short periods of playing or walking in large urban parks or urban woodland landscapes can enhance people's positive emotions^13^. Some scholars have found that viewing woodland and single-layer grassland landscapes has a more significant effect on people's physical and mental health than viewing other types of landscapes^14^. In a visual assessment of urban recreational landscapes, Polat et al. reported that the type of green space (e.g., water or plants) impacts visual quality, and it has also been mentioned that a lack of variation in the plant community type has a negative impact on visual quality^15^.

Walking in and seeing natural environments can enhance positive emotions and relieve stress^16,17^. Short stays in urban green spaces increase positive emotions and significantly relieve negative emotions that originate in developed environments^18^. Current research methods on landscape experience usually emphasise the public's enjoyment of the landscape in terms of visual sensory stimulation, and diverse methods, including photographic viewing, video viewing, and virtual reality (VR) technologies, have emerged for experiencing landscape types^19^. With the development of image technology, an increasing number of researchers have studied the convenience, ease of manipulation and ability to modify images while using photo, video, and VR technologies to present visual landscape experiences. Due to limitations associated with photographic and perceptual experiences, participants are unable to fully experience landscape features and capture feedback through multiple sensory responses, and the effectiveness of the landscape experience may be affected. Being at a location enables more direct and effective landscape perception, allowing individuals to experience the landscape more realistically and effectively^20^.

### Impact of seasonal changes on environmental perceptions

The natural environment consists of a variety of landscape elements, and the combination and layout of these different elements form diverse landscape environments. Green space is an important part of landscape environments, and these elements are associated with various vegetation types. The incorporation of plant community types in landscapes is important^21^. A combination of plant community types can beautify the environment and create space for recreation. People recognise that health can be improved by exposure to greenery; plant community types play a role in beautifying the environment and creating space^22^, and there is an increasing demand for space^23^. When people discovered the importance of green spaces for physical and mental health, an increasing number of people began to desire these spaces^24,25^. People in urban areas with less green space have poorer health, but they are likely to benefit more by spending more time in nature or living in areas with more green space^26^.

In addition, the appearance of green spaces may change considerably with the seasons, thus affecting people's perceptions and preferences. Different weather conditions during the same season can also affect preferences^27^. However, few studies have considered the effects of seasonal dynamics on landscape perception^28–30^. Most studies have introduced participants to landscapes in warmer seasons, such as spring and summer^31,32^. Song et al. reported that walking around and viewing urban parks and urban neighbourhoods in spring, autumn, and winter had positive physical and psychological restorative effects^33–35^. Bielinis E et al. reported that during the winter months, short exposure to natural environments enhanced positive emotions among participants^36^.

There are two major limitations of research on landscape perception and experience. First, some studies have shown that despite the positive effects of exposure to natural images, such natural exposure may be detrimental to human well-being in the long run^37,38^. Second, almost all of the existing studies were conducted in the summer months or used photographs of natural landscapes in the summer. Therefore, it is unclear whether the health benefits associated with contact with natural environments can also be obtained in colder seasons^39,40^. The winter season is a typical climatic season, and a concomitant decrease in the number of people participating in physical activity has an impact on people's outdoor activities, leading to a gradual decline in the extent to which people experience natural landscapes^41^; however, the desire to restore physical and mental health does not diminish in the winter. The lack of information about people's perceptions and preferences during the winter months limits the recreational quality of urban green spaces and potential improvements^40^. Therefore, it is necessary to compare visual perception strategies between the summer and winter seasons.

This study incorporated site perception approaches for both the winter and summer seasons and used two experiential forms: seated viewing and walking tours. Psychophysiological indicators such as skin conductance (EDA), ear-tip pulse (PPG), and the Perception of the Environment Scale (PRS) were used to monitor participants' reactions to viewing four plant community types (lawn, forest, forest-shrub-grass, and forest-grass) and plazas with no plants (the control group); additionally, physical and psychological recovery were monitored. The results of this study could inform strategies for designing planted landscapes in urban parks and assessing the perceptual experience of landscapes with plant communities, thereby establishing a scientific foundation for future landscape perception efforts.

Our study aimed to investigate the following questions:

1. What are the effects of perceiving two seasonal landscapes on participants' physical and psychological recovery?
2. How do different seasonal plant community landscapes affect participants' perceptual experiences?
3. What impact does the type of plant community have on participants' physiological recovery and emotional responses?

## Materials and methods

### Research area and objects

This study was conducted in Xi'an city (34°16′N, 108°54′E), the capital of Shaanxi Province, which has a total area of 10,752 either square kilometers and a population of 12.6 million. The area of green coverage in this city is approximately 33.5%^42^. In this study, a park in Xi'an with a landscape of plant communities and different vegetation structures was selected as the study area. Green spaces with coloured foliage were excluded to ensure that variations in perception and preference were due to vegetation structure rather than other landscape attributes.

In this study, green spaces were created with combinations of three levels of vegetation: trees, shrubs, and herbs.. The vegetation types include single layers that create an open green space (e.g., grassland, single-layer woodland), semiopen green space formed by combinations of trees and grass (e.g., understorey and overstorey landscapes), and closed green spaces created by combinations of trees, shrubs, and grasses.

**Figure 1:**
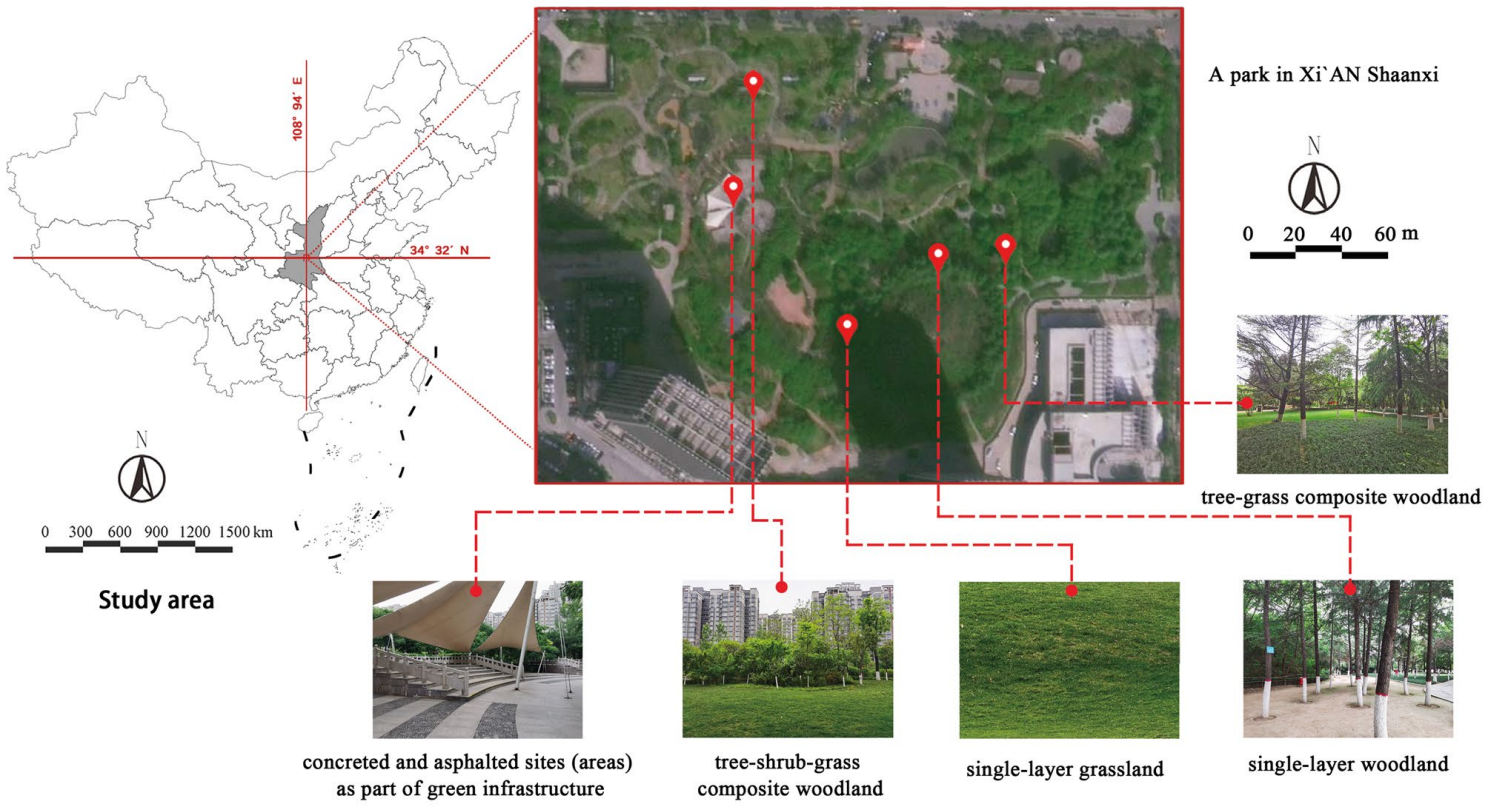
Study area. (Figure source: Base map from Autonavi Navigation; figure created by the first author. 2024 Autonavi Navigation—GS(2024)1158, and https://www.amap.com/search?query=%E6%96%87%E6%99%AF%E5%85%AC%E5%9B%AD&city=610100&geoobj=108.932943%7C34.320155%7C108.939129%7C34.322729&zoom=17.87).

**Figure 2:**
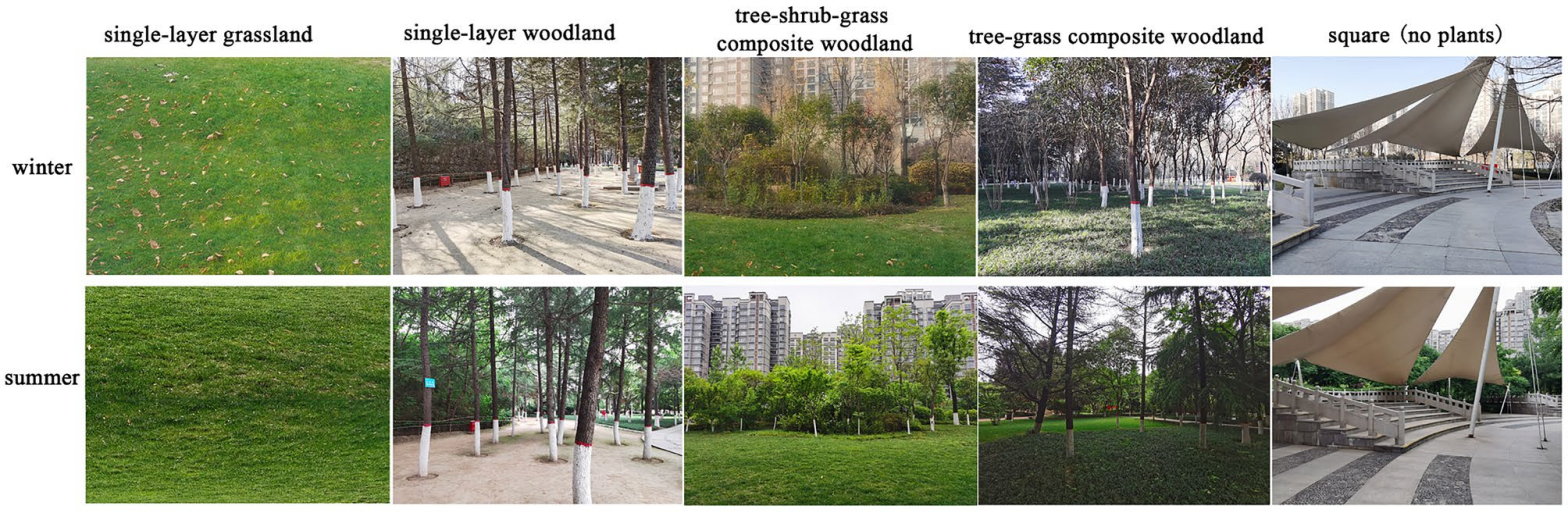
Research object. (Figure source: The image was created and photographed by the first author).

The morphologies and spatial attributes of the landscape spaces were used to distinguish the different vegetation structures; in total, four vegetation structures and a control group without plants were selected. The vegetation structures included single-layer grassland; single-layer woodland; tree-shrub-grass composite woodlands; and tree-grass composite woodland. Concrete squares without plants were present in the park, and the four vegetation structure types were chosen because they are common in Xi'an (Figs. 1 and 2, and Table 1).

**Table 1:** Different vegetation types in green spaces.

Vegetation types	Plant species
Single-layer grassland	*Axonopus compressus (Sw.) Beauv*
Single-layer woodland	*Pinus tabuliformis Carr*
Tree-shrub-grass composite woodlands	*Gleditsia sinensis Lam., Ligustrum lucidum Ait., Prunus cerasifera Ehrhar f. atropurpurea (Jacq.) Rehd., Nandina domestica, Euonymus japonicus 'Aureo-marginatus', and Axonopus compressus (Sw.) Beauv*
Tree-grass composite woodland	*(Gleditsia sinensis Lam. and Axonopus compressus (Sw.) Beauv*

### Test object

A total of 400 college students (M age = 21.85, SD age = 3.34, age range = 17–25 years) were recruited as participants in this study. All the participants were healthy students and spoke Chinese. All participants were informed about the experimental procedures, associated risks, and confidentiality issues, and all signed an informed consent form before the experiment. The study was conducted in accordance with the Declaration of Helsinki. Participants were randomly assigned to one of 10 groups, with each group consisting of 40 individuals and corresponding to a specific combination of vegetation type and season in green spaces.

### Physiopsychological indicators

#### Physiological measurement indicators

In this study, skin conductance (EDA) was used to monitor the skin conductance level (SCL) of the subjects and to record in real time the changes in physiological stress and comfort during the stress task and during the tour of different plant community types.

SCL has been shown to be positively correlated with sympathetic nerve activity in humans and is often used as a measure of stress; to a certain extent, SCL can reflect elevated levels of stress in individuals. Significant increases in SCL typically indicate a transition from a stable to a stressful state, while decreases suggest a move towards relaxation and calmness^43^.

#### Perceived restorativeness scale (PRS)

Kaplan summarised 4 attributes of restorative environments that can stimulate people's mental states and support recovery, namely, being away, coherence, fascination and compatibility^44^. Participants rated the characteristics of the four landscapes on a 5-point scale ranging from -2 to 2, with positive scores indicating better recovery from mental fatigue and negative scores suggesting increased mental fatigue. The PRS is currently considered a more general method for evaluating the degree of recovery from mental fatigue.

### Experimental design

#### Test procedures

We began by briefly explaining the experiment to the participants and obtaining their written informed consent. After completing a short demographic questionnaire, participants were taken to the test scenario area, where electrodes were attached to their skin to continuously monitor and measure their SCL throughout the trial. Participants were then asked to relax for 3 min. The mean SCL during this relaxation period was used as the baseline SCL for assessing the impacts of the test scenarios and was recorded as M0. Participants subsequently completed a 3-min mathematical test to induce stress^45^. The mean SCL during the test was used to represent the SCL of the participants under stress, after which they completed an initial PRS assessment. This stage was recorded as M1 Each participant was then randomly assigned to one of five scenes, in which participants were placed in a seated viewing position and asked to wear handmade glasses, which were used to limit the range of the participant's visual field. Participants were shown the scenes in a seated position for 3 min^36^, and their baseline mean SCL was measured and recorded. This phase was recorded as M2. After viewing the scene in a seated position, the participant took a 1-min break and again performed a walking tour, which included a slow walk around the scene. Following the walking tours of the scene, participants completed the final PRS assessment, which was recorded as M3. After removing the SCL measurement equipment, participants left the test site. The duration of the experiments ranged from 0.4 to 0.5 h.

(a) Winter sensory experience.

The winter trial, conducted from 1 to 30 November 2020, had an average temperature of 11.2 °C (11.2 ± 1.27), with clear skies throughout the trial period, and the weather was clear (no rain). In order to reduce the impact of interfering variables, we ensured that the surroundings were quiet and that the light, temperature, humidity, and wind speed were consistent across the landscape areas. Before the test began, notices were posted within 2 m of the test site to inform visitors of the ongoing study, thereby minimising potential interference from external factors such as visitors' activities and noise.

(b) Summer sensory experience.

The summer trial, conducted from 1 to 30 June 2021, had a mean temperature of 26.8 °C (26.8 ± 2.75), and the weather remained clear throughout the trial period. The summer trial employed the same protocol for controlling external confounding variables as the winter trial.

#### Stressful tasks

To induce stress, participants were asked to complete a 3-min mathematical test. We told participants that the purpose of this experiment was to assess their performance on the numerical calculations, and we scored and ranked their performance to reflect participants’ physiological recovery and emotional changes more clearly after the experience. We used numerical calculations and simulations of noisy environments to induce psychological and physiological responses. Previous studies have demonstrated the effectiveness of noisy environments as stressors through SCL or emotional responses, showing that stressors can increase SCL or suppress mood, respectively^46^.

### Data analysis

Data were processed using IMB SPSS Statistics 26.0 software (IBM, Inc., Armonk, NY, USA), and the changes in the relevant physiological and psychological indicators were calculated using the following formulas:

(1) Changes in physiological recovery at each stage.

Stress pressure value (△M1) = M1-M0,

Sitting excursion value (△M2) = M2-M0,

Walking tour value (△M3) = M3-M0,

Recovery value (△M4) = △M3 + △M2-△M1.

(2) PRS change value.

PRS change value (∆M`1) = M` before experience—M` after experience.

We tested the effects of the two perception methods and the four vegetation structure types on physical and mental recovery by comparing the recovery values (ΔM4) of the different vegetation structures using one-way ANOVA for each perception method. We assessed the impact of each vegetation structure type on perceived environmental restorativeness by conducting a paired-samples t test to compare dimension scores before and after the experience.

### Ethical statement

All subjects gave their informed consent for inclusion before they participated in the study. The study was conducted in accordance with the Declaration of Helsinki, and the protocol was approved by the Ethics Committee of the College of Architecture, Chang'an University.

## Results

### Effect of vegetation structure on participants' physiological recovery (SCL) across seasons

#### Effect of summer perception on participants' SCL

**Figure 3:**
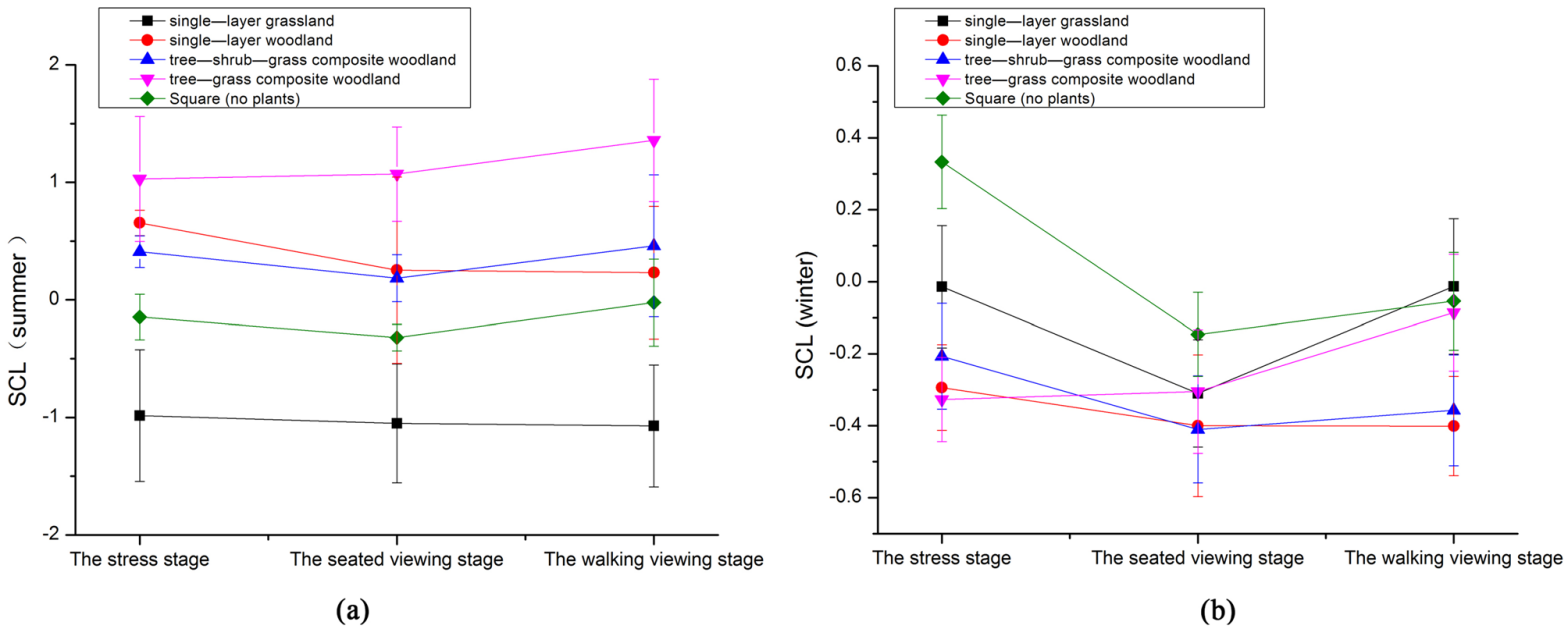
Effect of perceptual experience on SCL in different seasons ((**a**) Summer perception, (**b**) Winter perception).

During the summer, our study of green spaces with varying vegetation structures revealed a decreasing trend in participants' SCL values after viewing single-layer grassland, single-layer woodland, and single-storey woodland in the seated viewing phase, suggesting a gradual reduction in stress compared to the stress phase (ΔM1). Participants exhibited a significant decrease in SCL values after viewing single-storey woodland and tree-shrub-grass composite woodlands. During the walking tour stage (ΔM3), SCL values in participants who viewed single-layer grassland and single-layer woodland gradually declined, with a more significant decrease observed among those who viewed the single-layer woodland compared to the seated viewing stage. For participants who viewed tree-shrub-grass composite woodlands and concrete squares without plants, an increasing trend in SCL values was observed. Conversely, participants who viewed tree-grass composite woodlands experienced an increasing trend in SCL values throughout the experiment, indicating a gradual increase in stress. During the seated viewing phase, participants exposed to concrete squares without plants initially showed a brief decrease in SCL values, but these values gradually increased during the walking tour phase, leading to an overall increase in stress. Our findings indicate that viewing and walking in the single-layer woodland during the summer were associated with higher levels of stress, whereas the body appeared to be significantly more relaxed in the seated position (Fig. 3a).

#### Effects of winter perception on participants' SCL

Our study of green spaces with various vegetation structures in winter revealed that, compared to the stress phase (∆M1), participants' SCL values generally decreased during the seated viewing phase (∆M2) after being exposed to single-layer grassland, single-layer woodland, tree-shrub-grass composite woodlands, and concrete squares without plants. A significant decrease in SCL values was observed after participants viewed single-layer grassland, single-layer woodland, and concrete squares without plants. In the walking tour phase (ΔM3), participants' SCL values started to gradually decrease compared to the seated viewing phase. For the remaining landscape types (single-layer grassland, single-layer woodland, and concrete squares without plants), participants' SCL values exhibited an increasing trend. Throughout the experiment, participants who viewed tree-grass composite woodlands experienced an increase in SCL values. Participants' SCL values showed varying responses to concrete squares without plants between winter and summer. Viewing concrete and asphalt squares without plants resulted in a shorter relaxation period for participants, and prolonged exposure to this environment led to an increased stress response. In contrast, walking among single-layer woodlands in winter was more effective for stress relief and resulted in a significant and noticeable relaxation of the body (Fig. 3b).

### Effects of viewing green spaces with different vegetation structures on perceived environmental restorativeness among participants

#### Effect of summer perception on the perceived restorativeness of the environment

**Figure 4:**
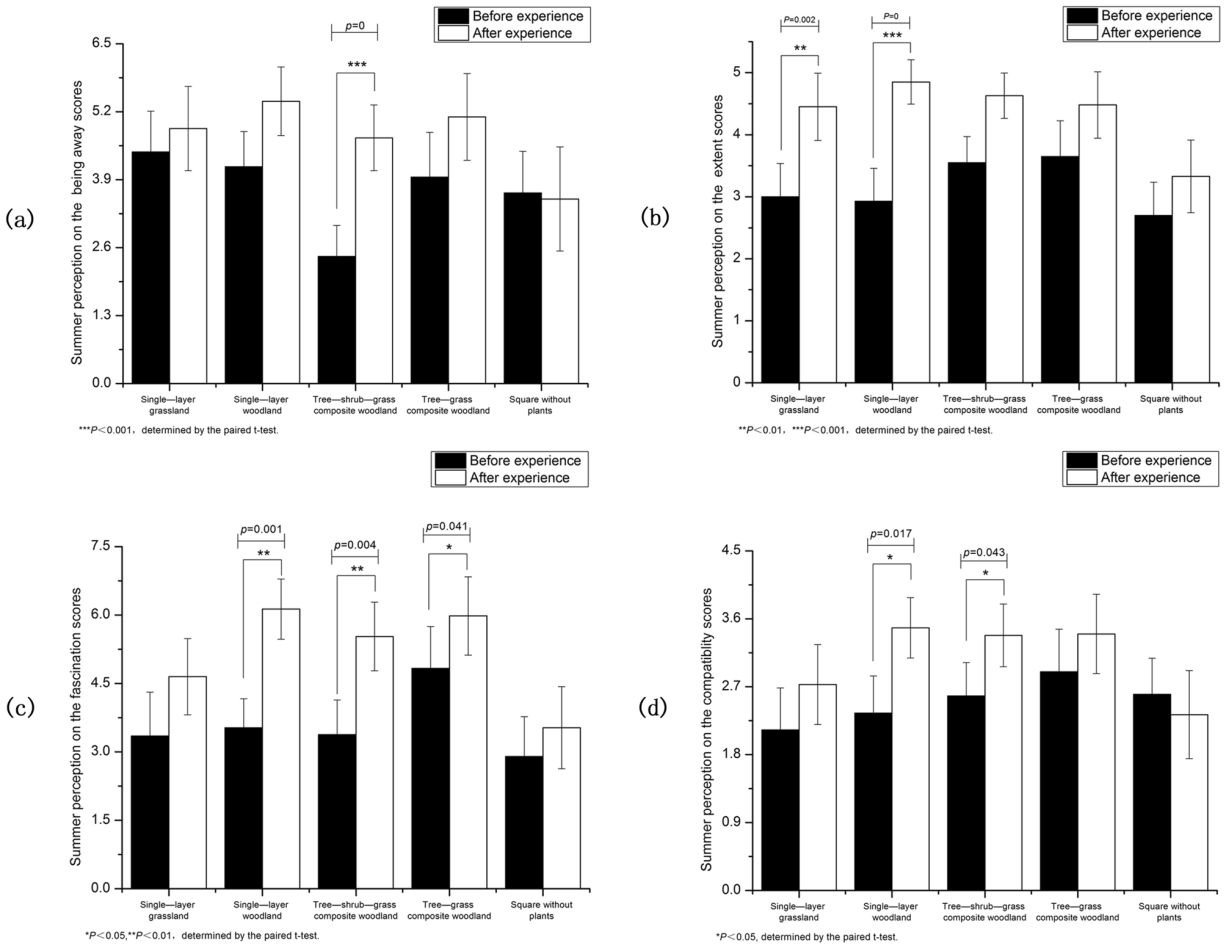
The impact of environmental perception on PRS scores in summer ((**a**) Being away score, (**b**) extend score, (**c**) fascination score, (**d**) compatibility score).

Paired t test results for the four dimensions of the environmental perception scale before and after exposure in the summer are presented in Fig. 4. Our analysis revealed that green spaces with different vegetation structures achieved higher postmeasurement scores on all four dimensions compared to concrete and asphalt squares without plants. Among the four vegetation structure types, single- layer woodland and tree-shrub-grass composite woodlands had higher scores for the four dimensions.

(1) Being away.

After viewing tree-shrub-grass composite woodlands and tree-grass composite woodlands, participants' being away scores were significantly higher than before exposure to green spaces (p < 0.05). Participants' being away scores also increased after experiencing single-layer grassland and single-layer woodland compared to their scores before exposure to the green spaces, but the increasing trend was smaller than that for tree-shrub-grass composite and tree-grass composite woodlands. The being away scores decreased when participants were exposed to concrete squares without plants. After participants experienced the different scenarios, their △M`1 values were ranked from largest to smallest as follows: tree-shrub-grass composite woodlands, tree-grass composite woodlands, single-layer woodland, single-layer grassland, concrete squares without plants (Fig. 4a).

(2) Extent.

The extent scores of all five scenario types showed an upward trend compared to the pre-experience scores, with significant differences observed for single-layer grassland, single-layer woodland, and tree-shrub-grass composite woodlands (p < 0.05). The concrete squares without plants had higher scores than the green spaces with different vegetation structures, while the latter had extent scores greater than those of the concrete squares without plants. After participants experienced the different scenarios, their △M`1 values were ranked from largest to smallest as follows: single-layer woodland, tree-shrub-grass composite woodlands, single-layer grassland, tree-grass composite woodlands, concrete squares without plants (Fig. 4b).

(3) Fascination.

Fascination scores generally increased after participants viewed the five landscape types compared to their preexposure scores, with significant differences observed for single-layer woodland, tree-shrub-grass composite woodlands, and tree-grass composite woodlands (p < 0.05). Fascination scores were higher for the concrete squares without plants than for the green spaces with different vegetation structures. After participants experienced the different scenarios, their △M`1 values were ranked from largest to smallest as follows: single-layer woodland, tree-shrub-grass composite woodlands, tree-grass composite woodlands, single-layer grassland, concrete squares without plants (Fig. 4c).

(4) Compatibility.

Compatibility scores generally increased after exposure to all four vegetation structures, whereas they tended to decrease for the concrete squares without plants. Compared to the pre-experience scores, the compatibility scores tended to increase after exposure to all four vegetation structures, while compatibility scores generally increased after exposure to all four vegetation structures, whereas they tended to decrease for the concrete squares without plants. Significant differences were observed between single-layer woodland and tree-shrub-grass composite woodlands before and after exposure (p < 0.05), with a more pronounced upwards trend in compatibility scores for these green spaces compared to single-layer grassland and tree-grass composite woodlands. After participants experienced the different scenarios, their △M`1 values were ranked from largest to smallest as follows: single-layer woodland, tree-shrub-grass composite woodlands, single-layer grassland, tree-grass composite woodlands, concrete squares without plants (Fig. 4d).

#### Effects of winter on perceptions of environmental restorativeness

**Figure 5:**
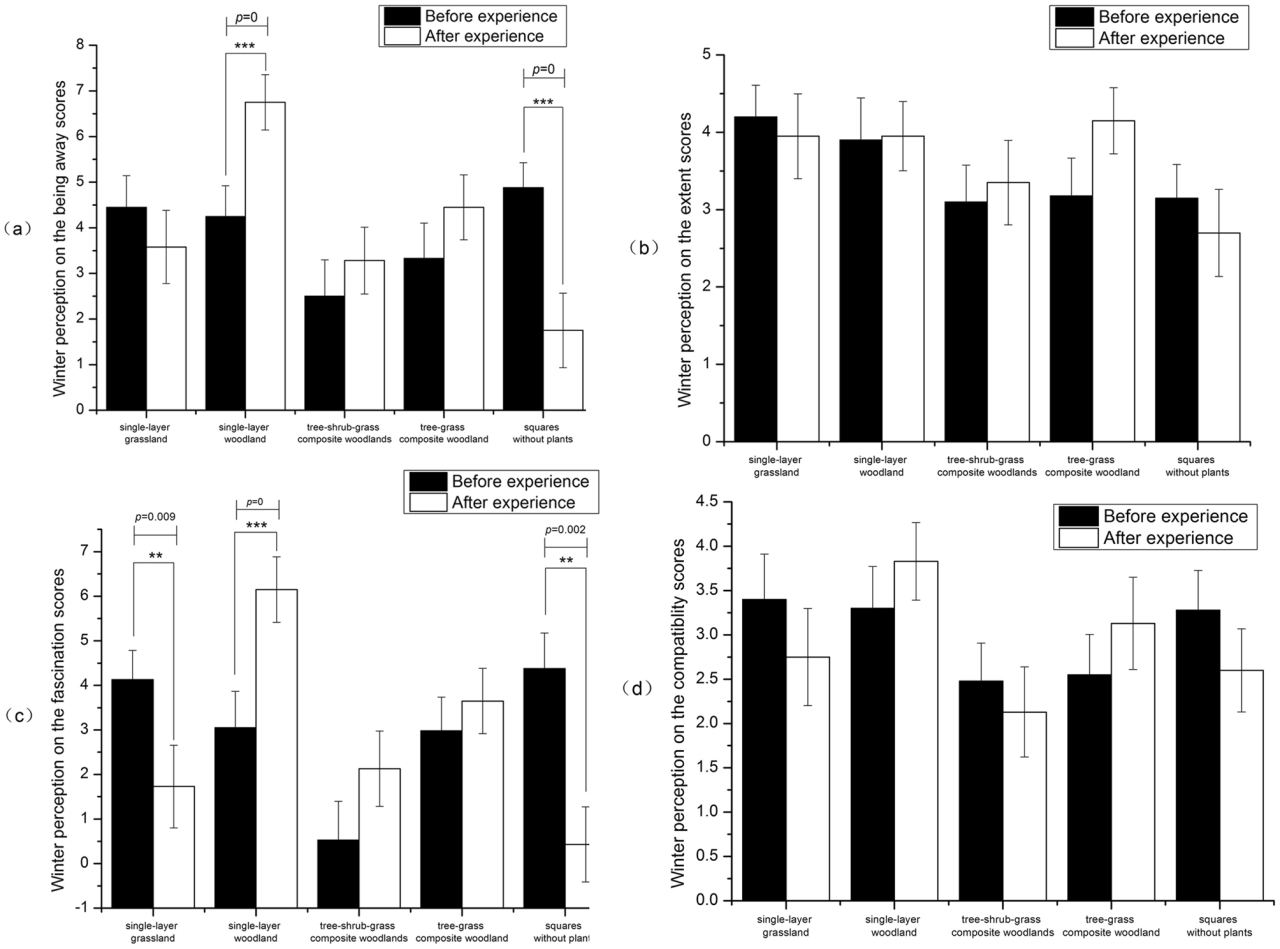
Impact of winter environmental perception on PRSs ((**a**) Being away score, (**b**) Extent score, (**c**) Fascination score, (**d**) Compatibility score).

The winter trial exhibited less variation in pre- and postexperience scores compared to the summer trial, a pattern closely associated with the outdoor physical environment in winter. Paired t test results for the pre- and postexperience measurements of the four dimensions of the environmental perception scale in the winter trial are presented in Fig. 5. Our analysis revealed that for all four dimensions, participants' scores were higher after experiencing green spaces with different vegetation structures compared to concrete and asphalt squares without plants.

(a) Being away.

Compared to the pre-experience scores (∆M` pre-experience), participants' being away scores (∆M` postexperience) tended to increase after viewing single-layer woodlands, tree-shrub-grass composite woodlands, and tree-grassed composite woodlands. The being away scores tended to decrease after participants experienced single-layer grassland and concrete squares without plants, with the highest being away scores occurring after participants experienced single-layer woodland. The being away scores decreased significantly after participants experienced concrete squares without plants. After participants experienced the different scenarios, their △M`1 values were ranked from largest to smallest as follows: single-layer woodland, tree-grass composite woodland, tree-shrub-grass composite woodlands, single-layer grassland, concrete squares without plants (Fig. 5a).

(b) Extent.

The participants' extent scores before and after experiencing the different scenarios were examined. The results revealed that significantly higher extent scores were observed after participants experienced the four vegetation structures compared to the concrete squares without plants. After experiencing the tree-grass composite woodland, participants rated the overall feeling of the scenario as better, followed by the tree-shrub-grass composite woodlands and single-layer woodland, while they reported the worst overall feeling after viewing the single-layer grassland. After participants experienced the different scenarios, their △M`1 values were ranked from largest to smallest as follows: tree-grass composite woodlands, tree-shrub-grass composite woodlands, single-layer woodland, single-layer grassland, concrete squares without plants (Fig. 5b).

(c) Fascination.

Participants' fascination scores were significantly higher after experiencing the green spaces with different vegetation structures than after experiencing the concrete squares without plants, as indicated by our analysis of the pre- and postexperience ratings. Compared with the pre-experience rating (ΔM` before the experience), participants rated the scenarios as more favourable after viewing the single-layer woodland, tree-shrub-grass composite woodlands, and tree-grass composite woodlands; participants’ fascination scores decreased significantly after experiencing the single-layer grassland and concrete squares without plants. such as "Significant differences were found in the fascination scores before and after experiencing single-layer woodlands (p < 0.001), single-layer grassland (p < 0.01), and concrete squares without plants (p < 0.001). After participants experienced the different scenarios, their △M`1 values were ranked from largest to smallest as follows: single-layer woodland, tree-shrub-grass composite woodlands, tree-grass composite woodlands, single-layer grassland, concrete squares without plants (Fig. 5c).

(d) Compatibility.

Analyses of participants' compatibility scores after experiencing the different experimental scenarios showed that, compared to the scores after experiencing concrete squares without plants, participants' compatibility scores after experiencing the single-layer woodlands and tree-grass composite woodlands were significantly greater, while the scores decreased after experiencing tree-shrub-grass composite woodlands and single-layer grassland. The compatibility scores showed an increasing trend after participants experienced single-storey woodland and tree-grass composite woodland, and a decreasing trend was observed after participants experienced single-storey grassland. Compatibility scores decreased significantly after participants experienced concrete squares without plants. In comparison to the pre-experience scores (△M` pre-experience), the compatibility scores of the participants after experiencing the single-layer woodland (△M` postexperience) were greater. After participants experienced the different scenarios, their △M`1 values were ranked from largest to smallest as follows: single-layer woodland, tree-grass composite woodlands, tree-shrub-grass composite woodlands, single-layer grassland, concrete squares without plants (Fig. 5d).

### Seasonal variation and the effect of green spaces with different vegetation structures on physiological recovery and perceived environmental resilience

#### Seasonal variation

Our one-way ANOVA results revealed a significant difference in perceived recovery values between the environment in summer and winter, with significant differences observed only in the Perceived Restorativeness Scale (PRS) for extent and compatibility (Table 2). Environmental perception scores for extent and compatibility were higher in summer compared to winter. Our findings suggest that the natural environment in summer is more conducive to restorative experiences for participants compared to winter.

**Table 2:** Changes in participants' physiological recovery and environmental perception scores after viewing green spaces with vegetation structures (excluding concrete squares without plants) in different seasons.

	Summer	Winter	F	Significance (*P*)
SCL	0.156 ± 0.541	− 0.36 ± 0.203	0.799	0.372
Being away	1.28 ± 0.316	0.84 ± 0.359	0.837	0.361
**Extent**	**1.32 ± 0.217**	**0.11 ± 0.217**	**14.69**	**0**
Fascination	1.8 ± 0.354	0.74 ± 0.475	3.18	0.075
**Compatiblity**	**0.76 ± 0.201**	**0.03 ± 0.217**	**5.039**	**0.025**

Significant values are in bold.

#### Effects of green spaces with different vegetation structures on physiological recovery and perceived environmental restorativeness

Our analyses revealed that vegetation type had a significant impact on recovery and emotional responses (being away and fascination) in green spaces, but not on Skin Conductance Level (SCL), as shown in Table 3.

**Table 3:** Effects of physiological recovery and emotional responses experienced in green spaces with different types of vegetation structures (containing concrete squares without plants).

	Single-layer grassland	Single-layer woodland	Tree-shrub-grass composite woodland	Tree-grass composite woodland	The concrete and asphalt space	F	Significance (*P*)
SCL	− 0.72 ± 0.85	− 0.19 ± 0.47	− 0.16 ± 0.34	0.67 ± 0.53	− 0.37 ± 0.27	0.93	0.45
**Being away**	** − 0.29 ± 0.13**	**1.88 ± 0.45**	**1.53 ± 0.44**	**1.14 ± 0.46**	** − 1.63 ± 0.38**	**8.68**	**0**
Extent	0.6 ± 0.34	0.99 ± 0.34	0.66 ± 0.28	0.6 ± 0.33	0.09 ± 0.04	0.97	0.43
**Fascination**	** − 0.55 ± 0.13**	**2.85 ± 0.5**	**1.88 ± 0.36**	**0.91 ± 0.22**	** − 1.66 ± 0.44**	**8.69**	**0**
Compatiblity	− 0.03 ± 0.07	0.83 ± 0.29	0.23 ± 0.33	0.54 ± 0.32	− 0.48 ± 0.14	2.32	0.06

Significant values are in bold.

Significant differences in being away scores were observed in only five cases, specifically between single-layer grassland and single-layer woodland (p = 0.002), single-layer woodland and tree-shrub-grass composite woodlands (p = 0.01), single-layer grassland and tree-grass composite woodlands (p = 0.042), single-layer woodland and concrete squares without plants (p = 0), tree-shrub-grass composite woodlands and concrete squares without plants (p = 0), and tree-grass composite woodlands and concrete and asphalt squares without plants (p = 0). Among the green spaces evaluated, single-layer woodlands exhibited the highest recovery value, followed by tree-shrub-grass composite woodlands, tree-grass composite woodlands, single-layer grassland, and concrete squares without plants.

Only six comparisons showed significant differences in scores: between single-layer grassland and single-layer woodland (p = 0), single-layer grassland and tree-shrub-grass composite woodlands (p = 0.006), single-layer woodland and tree-grass composite woodlands (p = 0.027), tree-shrub-grass composite woodlands and concrete squares without plants (p = 0), single-layer woodland and concrete squares without plants (p = 0), and tree-grass composite woodland and concrete squares without plants (p = 0.003). Among the green spaces assessed, single-layer woodlands demonstrated the highest recovery value, followed by tree-shrub-grass composite woodlands, tree-grass composite woodlands, single-layer grassland, and concrete squares without plants.

#### Effects of seasonal changes in green spaces with different vegetation structures on participants' physiological recovery and perceptions of environmental resilience

(1) Effects of seasonal changes in green spaces with different vegetation structures on participants' physiological recovery.

**Figure 6:**
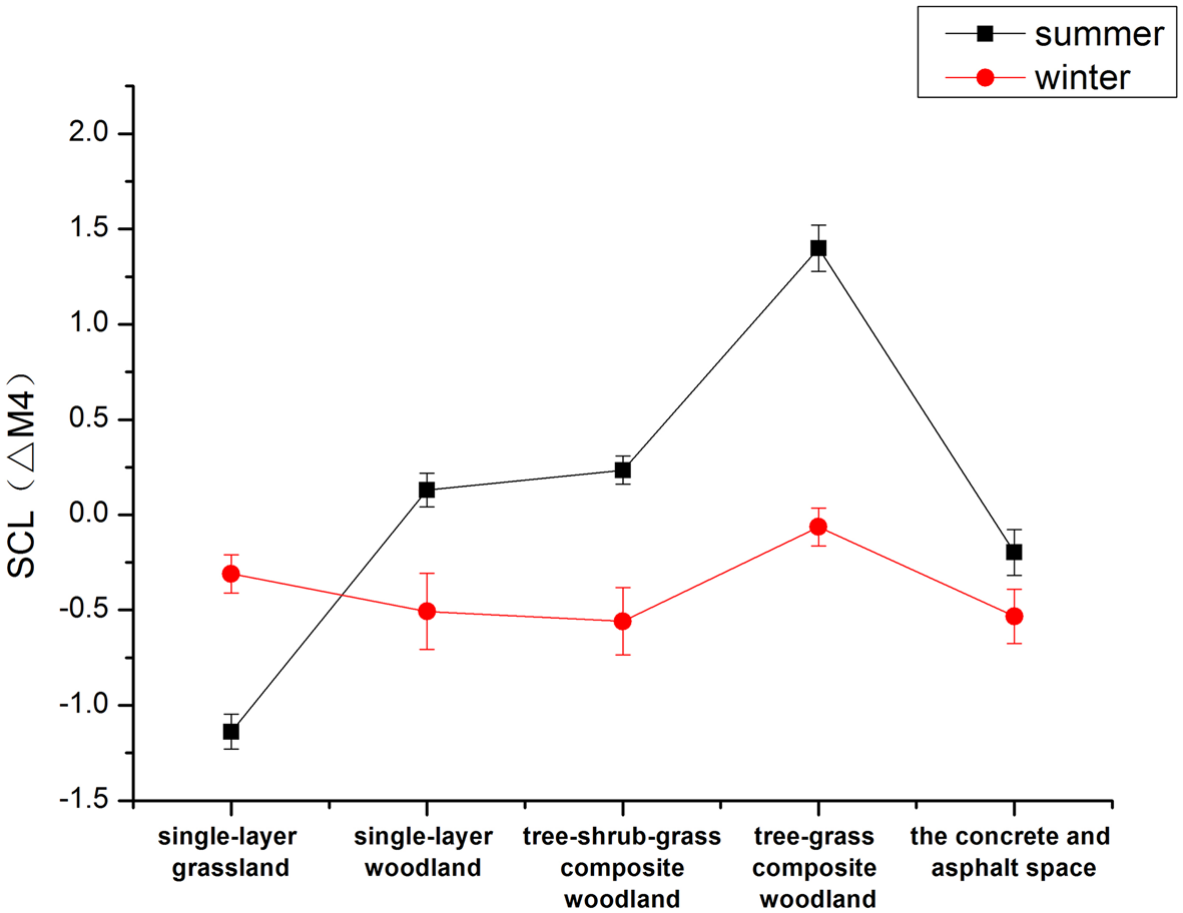
Seasonal changes in green spaces with different vegetation structures on participants' SCLs.

Our paired t tests revealed a significant increase in participants' physiological recovery values (ΔM4) in summer when viewing single-layer woodlands, tree-shrub-grass composite woodlands, and tree-grass composite woodlands, compared to their physiological recovery values in winter. Notably, participants' physiological recovery values (ΔM4) showed a significant increase after viewing the tree-grass composite woodland in summer. Regardless of season, viewing tree-grass composite woodlands consistently had a positive impact on participants' physiological recovery, as illustrated in Fig. 6.

(2) Effect of seasonal changes in green spaces with different vegetation structures on the perception of environmental restorativeness.

**Figure 7:**
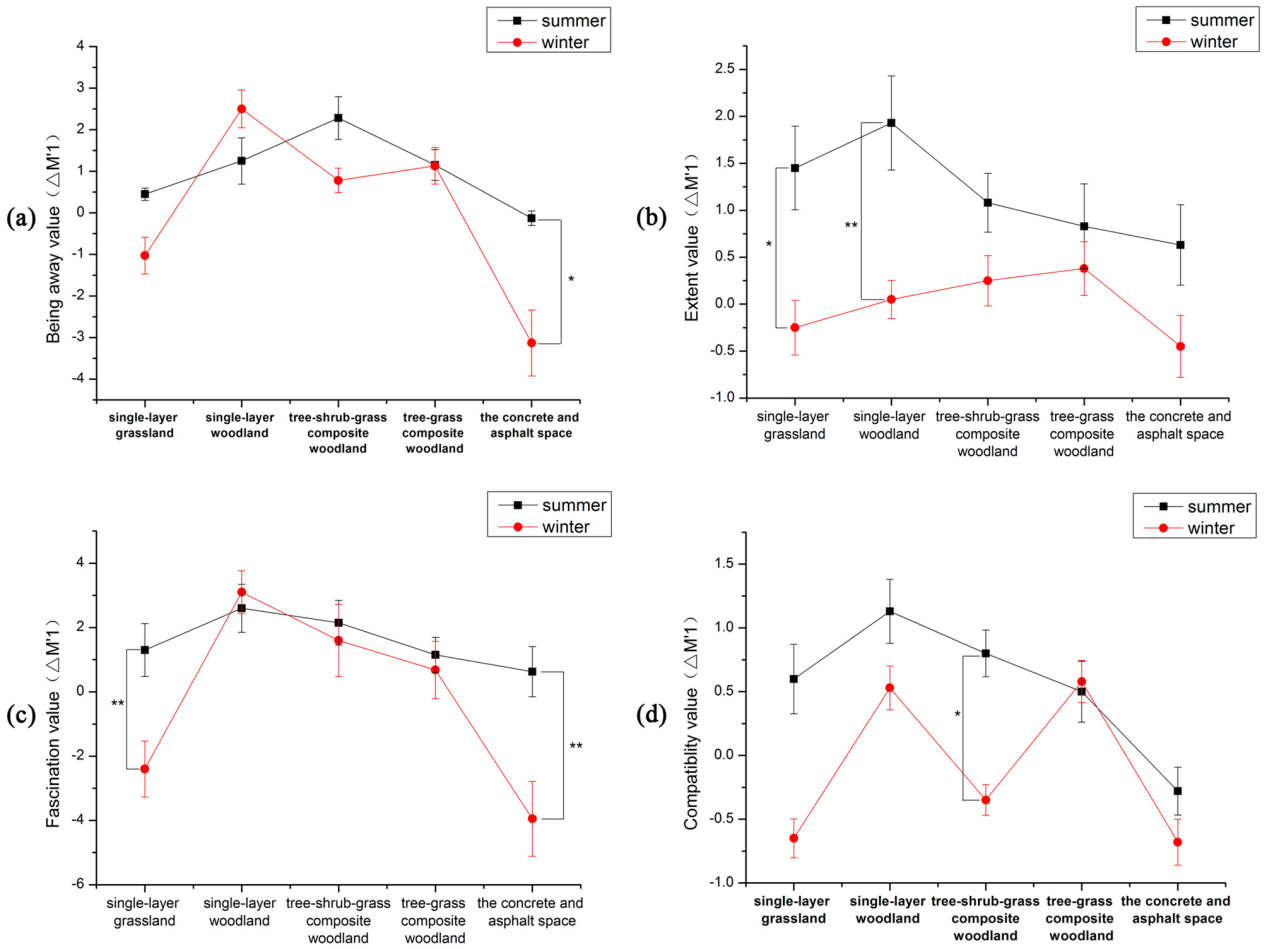
The effect of seasonal changes in green spaces with different vegetation structures on participants' PRSs.

PRSs for green spaces with different vegetation structures were generally lower in winter than in summer, reflecting the influence of the outdoor physical environment on these perceptions. Figure 7 shows the results of our investigation into the four dimensions of the PRSs for green spaces with different vegetation structures in both summer and winter. Our results indicate that green spaces with different vegetation structures were perceived differently across the four dimensions of the PRSs in winter and summer, and in both seasons, PRSs for green spaces with vegetation were higher than for concrete squares without plants.

(a) Being away.

The being away scores were significantly higher for concrete squares without plants in the summer compared to the winter (p = 0.012), but there were no significant seasonal differences in being away scores for green spaces with different vegetation structures. However, the being away scores were greater for the single-layer woodland in the winter than for the single-layer woodland in the summer (Fig. 7a).

(b) Extent.

After experiencing single-layer grassland and single-layer woodland in the summer, participants had significantly higher extent scores than when they experienced these landscapes in the winter. Specifically, the single-layer woodland received the highest ratings, followed closely by the single-layer grassland, with p-values of 0.005 and 0.02, respectively (Fig. 7b).

(c) Fascination.

We analyzed the fascination scores of participants who experienced green spaces with different vegetation structures in various seasons. Our results revealed that participants had significantly higher fascination scores in the summer for these green spaces than in the winter. In particular, participants enjoyed the scenes more after viewing the single-layer grassland (p = 0.01) or concrete squares without plants (p = 0.002) in the summer. Moreover, the fascination scores for single-layer woodland in the winter were higher than those in the summer (Fig. 7c).

(d) Compatibility.

Analysis of compatibility scores revealed that participants who experienced tree-shrub-grass composite woodlands in the summer had significantly higher compatibility scores than those who experienced green spaces with different vegetation structures in the winter. In contrast, participants who experienced tree-grass composite woodlands in the winter had slightly higher compatibility scores than those who experienced green spaces with different vegetation structures in the summer (Fig. 7d).

## Discussion

### Effects of different seasons on physiological recovery and environmental perceptual resilience among participants

Environmental perception varied significantly between seasons, indicating that exposure to natural environments in summer was more beneficial for participants. Significant differences in participants' physiological recovery between seasons were mainly attributed to the restorative aspects of environmental perception (being away, extent, compatibility), and no significant differences were observed in physiological recovery. Exposure to natural environments during winter led to a decrease in participants' SCL values, whereas in summer, such exposure resulted in an increase in their extent and fascination scores, indicating that summer landscapes were more effective at relaxing and soothing participants' emotions compared to winter landscapes, which had a less pronounced effect on reducing stress levels. Landscape perceptions can be significantly affected by seasonal variables, potentially because changes in plant biology during seasonal transitions alter the visual and ecological characteristics of vegetation, such as color, shape, density, and biodiversity, which in turn influence visual perceptions and psychological responses^27,47^. The established relationship between colour-induced visual stimulation and psychological functioning suggests that leaf colour variations across different landscape types, particularly forests, have a significant impact on landscape aesthetics and human perceptual and physiological states^48–51^. Therefore, plant species within the vegetation cycle (summer) and outside the vegetation cycle (winter) were considered, and the leaf colour of the selected plant species was considered green.

Seasonal changes primarily affect the human body through alterations in the physical environment, including temperature, humidity, and light. Significant differences in human comfort arise from temperature variations between winter and summer, with the main impact being on the body's ability to recover from mental fatigue, as evidenced by the scores on the environmental perception dimensions. When evaluating their ability to recover from mental fatigue, participants will independently assess green spaces with varying vegetation structures, taking into account not only sensory factors but also the influence of the surrounding environment, temperature, and humidity. People experience a greater sense of relaxation in summer landscapes, leading to enhanced abilities to recover from mental fatigue compared to winter. Conversely, the bleakness of winter landscapes tends to diminish people's ability to recover from mental fatigue^52^. As a result, the perceptual experience of summer landscapes is more profound than that of winter landscapes.

### Seasonal changes in green spaces with different vegetation structures affect participants' landscape experiences

Concrete squares without plants elicited lower PRSs, likely due to the hard material's reminder of indoor environments where participants spend much of their time working and living. In summer, the PRSs for concrete squares without plants were higher than in winter, a phenomenon attributed to the vibrant colours and diverse vegetation structures present in the surroundings^53^. While the PRSs for concrete squares without plants were higher in summer than in winter, the overall perception of environmental restoration was not significantly favoured by participants. Further research is required to ascertain if enhancing the planted landscape around concrete squares without plants in future landscape designs could significantly boost the restorative effect of environmental perception. Seasonal variations in PRSs among participants may be influenced by the monotony of single-layer grassland, with higher scores reported in summer and lower scores in winter. Our findings indicate that the restorative power of single-layer grassland is primarily driven by visual comfort, encompassing the impacts of the landscape and sunlight on individuals' perceptions^54^. Research has demonstrated that seasonal changes can impact people's connection with nature in urban settings^55^, and some studies have posited that winter may negatively affect people's well-being indices^56^. Walking in urban parks during winter has been found to boost positive emotions^33^; yet people's perception of environmental restorativeness remains largely based on subjective feelings, and single-layer grasslands do not adequately shield against the cold in winter.

Tree-grass composite woodlands showed comparable PRSs in winter and summer, aligning with previous research on seasonal preferences^57^. This may be due to the attractiveness of the tree-grass composite woodlands. The tree-grass composite woodlands have a positive aesthetic quality, with a calm and relaxing atmosphere but a low level of approachability, which prevents participants from entering the space to experience and feel the landscape. Landscape preference is influenced by several perceptual scales, including spaciousness, naturalisation, species richness, and shelter^58^. In winter, single-layer woodlands scored higher on 'being away' and 'fascination' compared to summer, while in summer, they received higher ratings for 'extent'. The differences were statistically significant (p = 0.01). The visual characteristics of plants in different seasons affect landscape perception^59^; these characteristics include physical factors, such as the angle of incoming radiation, which is much less direct in winter than in summer and creates dappled shadows in single-layer woodlands, which enriches landscape interest and aesthetics. Tree shading is known to decrease surface temperatures and enhance latent heat exchange through evapotranspiration^60^. Beyond visual appreciation, quietness stands out as the most cherished attribute of green spaces, closely followed by spaciousness^61^, and single-layer woodland environments in winter are relatively quiet, allowing for a sense of relaxation. Additionally, the composition of plant species in single-layer woodlands is predominantly pine and cypress, and these trees release volatile organic compounds that have a calming effect on human emotions and physiology^49^. This observation underscores the seasonal differences in 'being away', 'fascination', and 'extent' within this type of landscape. PRSs for tree-shrub-grass composite woodlands were consistently higher during summer compared to winter, indicating that a balance of openness, moderate vegetation density, and biodiversity is essential to enhance the appeal of these landscapes^62^, fulfilling the participants' desires for scenic views and a sense of security^63^. Compared to single-layer woodlands, tree-shrub-grass composite woodlands exhibit a greater density. While this can sometimes hinder visibility and perceived safety, it also enhances privacy and provides ample shade, leading to higher PRSs in summer.

## Conclusions

In this study, we monitored participants' physiological recovery and psychological well-being based on their perception and experience of green spaces with different seasonal vegetation structures. This study aims to establish a scientific foundation for understanding how landscape perception and experience impact individuals and to serve as a theoretical guide for future landscape planning. Significant differences in perception were observed between the two seasons, with participants exposed to the summer landscape showing reduced SCL values and those in the winter landscape exhibiting higher scores for extent and compatibility. Second, exposure to single-layer woodlands and tree-shrub-grass composite woodlands in summer, as well as single-layer woodlands in winter, resulted in restorative effects for participants. Next, among the vegetation types studied, single-layer woodlands provided the most positive perceptual experience. In terms of perceptual experience, tree-shrub-grass composite woodlands came second, followed by tree-grass composite woodlands, single-layer grassland, and finally, concrete squares without vegetation. Lastly, the findings emphasize that these factors are distinct and not interchangeable. It is advisable for future landscape design to take into account the seasonal variations in vegetation when selecting plant species. Evergreen single-layer woodlands would be an ideal choice for winter urban landscapes.

This study has several limitations. First, all the participants were university students, so the results may not reflect other social groups. These results should be validated in other groups. In addition, the age range of the participants was between 17 and 25 years, and the results may differ for individuals of other ages. The age of the participants can be used as a variable in future research. Second, while the four vegetation types included in this study are common and representative of green spaces, they do not cover the full spectrum of possible vegetation structures. For a more comprehensive understanding of how different vegetation structures affect individuals, it is necessary to investigate additional vegetation types or plant species. Lastly, since the research was limited to winter and summer, the participants' preferences might have been biased by seasonal influences. Future research could extend this work to include all four seasons for a more complete comparison.

### Supplementary Information

Supplementary Information.

## Data Availability

The datasets generated and/or analysed during the current study are not publicly available due [The research data included in the manuscript is a subset of the data from Yifan Duan, the first author. To ensure that the use of the remaining research data and the publication of subsequent papers are not impacted, some of the data from this manuscript have been uploaded to the system as Supplementary Materials]. But are available from the corresponding author or first author on reasonable request. But are available from the corresponding author on reasonable request. The data was uploaded in the form of Supplementary Materials.

## References

[1] Wang Y, Lai J, Hu C, Meng H, Lyu D, Hu S (2021). Non-suicidal self-harm is linked to suicidal thoughts in Chinese adolescents with mood disorders: A cross-sectional report. J. Zhejiang Univ. Sci. B.

[2] Yao Y, Huang Q, Li S (2018). Study on the relationship between green space around workplace and physical and mental health: IT professionals in Beijing as target population. Chin. Landscape Architect..

[3] Lanki T, Siponen T, Ojala A, Korpela K, Pennanen A, Tiittanen P, Tsunetsugu Y, Kagawa T, Tyrväinen L (2017). Acute effects of visits to urban green environments on cardiovascular physiology in women: A field experiment. Environ. Res..

[4] Votsi NEP, Mazaris AD, Kallimanis AS, Drakou EG, Pantis JD (2014). Landscape structure and diseases profile: Associating land use type composition with disease distribution. Int. J. Environ. Health Res..

[5] Bertram C, Rehdanz K (2015). The role of urban green space for human well-being. Ecol. Econ..

[6] Deng W (2006). Landscape perception: Towards landscape semiology. World Architect..

[7] Shafer, Jr. E.L.; Hamilton Jr. J.F.; Schmidt, E.A. Natural landscape preferences: a predictive model. J. Leisure Res. 1, l–19 (1969).

[8] Zube EH, Sell JL, Taylor JG (1982). Landscape perception: Research, application and Theory. Landsc. Plan..

[9] Van den Berg AE, Jorgensen A, Wilson ER (2014). Evaluating restoration in urban green spaces: Does setting type make a difference?. Landsc. Urban Plan..

[10] Jim CY, Chen WY (2006). Perception and attitude of residents toward urban green spaces in Guangzhou (China). Environ. Manag..

[11] Sevenant M, Antrop M (2011). Landscape representation validity: A comparison between on-site observations and photographs with different angles of view. Landsc. Res..

[12] Simson S, Straus M (1997). Horticulture as Therapy: Principles and Practice.

[13] Tyrväinen L, Ojala A, Korpela K, Lanki T, Tsunetsugu Y, Kagawa T (2014). The influence of urban green environments on stress relief measures: A field experiment. J. Environ. Psychol..

[14] Falk JH, Balling JD (2010). Evolutionary influence on human landscape preference. Environ. Behav..

[15] Polat AT, Akay A (2015). Relationships between the visual preferences of urban recreation area users and various landscape design elements. Urban For. Urban Green..

[16] Song C, Ikei H, Kobayashi M, Miura T, Li Q, Kagawa T, Kumeda S, Imai M, Miyazaki Y (2016). Effects of viewing forest landscape on middle-aged hypertensive men. Urban For. Urban Green..

[17] Leggett AJ (2001). Bose-Einstein condensation in the alkali gases: Some fundamental concepts. Rev. Mod. Phys..

[18] Kang Y, Kim EJ (2019). Differences of restorative effects while viewing urban landscapes and green landscapes. Sustainability.

[19] Tong QI, Wang Y, Wang W (2013). A review on visual landscape study in foreign countries. Progress Geogr..

[20] Kjellgren A, Buhrkall H (2010). A comparison of the restorative effect of a natural environment with that of a simulated natural environment. J. Environ. Psychol..

[21] Yan H, Wang X, Hao P, Dong L (2012). Study on the microclimatic characteristics and human comfort of park plant communities in summer. Proc. Environ. Sci..

[22] Carpenter M (2013). From ‘healthful exercise’ to ‘nature on prescription’: The politics of urban green spaces and walking for health. Landscape Urban Plan..

[23] Lin W, Chen QB, Jiang M, Zhang X, Liu Z, Tao J, Wu L, Xu S, Kang Y, Zeng Q (2019). The effect of green space behaviour and per capita area in small urban green spaces on psychophysiological responses. Landscape Urban Plan..

[24] Wilson EO (1984). Biophilia.

[25] Vujcic M, Tomicevic-Dubljevic J, Zivojinovic I, Toskovic O (2019). Connection between urban green areas and visitors’ physical and mental well-being. Urban For. Urban Green..

[26] Cox DT, Shanahan DF, Hudson HL, Fuller RA, Gaston KJ (2018). The impact of urbanisation on nature dose and the implications for human health. Landscape Urban Plan..

[27] Półrolniczak M, Potocka I, Kolendowicz L, Rogowski M, Kupiński S, Bykowski A, Młynarczyk Z (2019). The impact of biometeorological conditions on the perception of landscape. Atmosphere.

[28] Junge X, Schüpbach B, Walter T, Schmid B, Lindemann-Matthies P (2015). Aesthetic quality of agricultural landscape elements in different seasonal stages in Switzerland. Landscape Urban Plan..

[29] Palang H, Fry G, Jauhiainen JS, Jones M, Sooväli H (2005). landscape and seasonality—Seasonal landscapes. Landscape Res..

[30] Stobbelaar DJ, Hendriks K, Stobbelaar DJ (2007). Seasonality of agricultural landscapes: reading time and place by colours and shapes. Seasonal landscapes.

[31] Berman MG, Jonides J, Kaplan S (2008). The cognitive benefits of interacting with nature. Psychol. Sci..

[32] Louv R (2011). The nature principle: human restoration and the end of nature deficit disorder. Child. Youth Environ..

[33] Song C, Joung D, Ikei H, Igarashi M, Aga M, Park BJ, Miwa M, Takagaki M, Miyazak Y (2013). Physiological and psychological effects of walking on young males in urban parks in winter. J. Physiol. Anthropol..

[34] Song C, Ikei H, Igarashi M, Miwa M, Takagaki M, Miyazaki Y (2014). Physiological and psychological responses of young males during spring-time walks in urban parks. J. Physiol. Anthropol..

[35] Song C, Ikei H, Igarashi M, Takagaki M, Miyazaki Y (2015). Physiological and psychological effects of a walk in urban parks in fall. Int. J. Environ. Res. Public Health.

[36] Bielinis E, Takayama N, Boiko S, Omelan A, Bielinis L (2018). The effect of winter forest bathing on psychological relaxation of young Polish adults. Urban For. Urban Green..

[37] Kahn PH, Severson RL, Ruckert JH (2009). The human relation with nature and technological nature. Curr. Direct. Psychol. Sci..

[38] Levi D, Kocher S (1999). Virtual nature: The future effects of information technology on our relationship to nature. Environ. Behav..

[39] Jung SG, Shin JY, Kum KT, Choi CH (2012). Sensibility image and preference analysis of street tree species using 3d simulation-focused on tongdaeguro in daegu metropolitan city. J. Korean Soc. Precis. Eng..

[40] Brooks AM, Ottley KM, Arbuthnott KD, Sevigny P (2017). Nature-related mood effects: Season and type of nature contact. J. Environ. Psychol..

[41] Gatti E, Brownlee M, Bricker KS (2021). Winter recreationists' perspectives on seasonal differences in the outdoor recreation setting. J. Outdoor Recreat. Tour..

[42] XABS, 2020. Statistical Bulletin of Xi’an 2019 National Economic and Social Development, 2. Bulletin of Xi`an People’ s Government, pp. 38–44. http://www.xa.gov.cn/gk/zcfg/zfgb/2020ndeq/tjsj/5ec38baff99d651fbf285b55.html (Accessed 11 November 2021).

[43] Braithwaite JJ, Watson DG, Jones R, Rowe M (2013). A guide for analysing electrodermal activity (EDA) & skin conductance responses (SCRs) for psychological experiments. Psychophysiology.

[44] Kaplan R, Kaplan S (1989). The Experience of Nature: A Psychological Perspective.

[45] Huang Q, Yang M, Jane HA, Li S, Bauer N (2020). Trees, grass, or concrete? The effects of different types of environments on stress reduction. Landscape Urban Plan..

[46] Markus CR, Panhuysen G, Tuiten A, Koppeschaar H, Fekkes D, Peters ML (1998). Does carbohydrate-rich, protein-poor food prevent a deterioration of mood and cognitive performance of stress-prone subjects when subjected to a stressful task?. Appetite.

[47] Kothencz G, Kolcsár R, Cabrera-Barona P, Szilassi P (2017). Urban green space perception and its contribution to well-being. Int. J. Environ. Res. Public Health.

[48] Zhang H, Tang Z (2011). To judge what color the subject watched by color effect on brain activity. Int. J. Comput. Sci. Netw. Secur..

[49] Ou LC, Luo MR, Woodcock A, Wright A (2004). A study of colour emotion and colour preference. Part I: Colour emotions for single colours. Color Res. Appl..

[50] Ou LC, Luo MR, Sun PL, Hu NC, Chen HS (2012). Age effects on colour emotion, preference, and harmony. Color Res. Appl..

[51] Kim JY, Lee HS (2009). A study on interior wall color based on measurement of emotional responses. Sci. Emot. Sensibil..

[52] Wang Y, Xu M (2021). Electroencephalogram application for the analysis of stress relief in the seasonal landscape. Int. J. Environ. Res. Public Health.

[53] Keniger LE, Gaston KJ, Irvine KN, Fuller RA (2013). What are the benefits of interacting with nature?. Int. J. Environ. Res. Public Health.

[54] Geng Y, Hong B, Du M, Yuan T, Wang Y (2022). Combined effects of visual-acoustic-thermal comfort in campus open spaces: A pilot study in China's cold region. Build. Environ..

[55] Duffy S, Verges M (2010). Forces of nature affect implicit connections with nature. Environ. Behav..

[56] Nisbet EK, Zelenski JM (2011). Underestimating nearby nature: Affective forecasting errors obscure the happy path to sustainability. Psychol. Sci..

[57] Duan Y, Li S (2022). Study of different vegetation types in green space landscape preference: Comparison of environmental perception in winter and summer. Sustainability.

[58] Grahn P, Stigsdotter UK (2010). The relation between perceived sensory dimensions of urban green space and stress restoration. Landscape Urban Plan..

[59] Kuper R (2013). Here and gone the visual effects of seasonal changes in plant and vegetative characteristics on landscape preference criteria. Landscape J..

[60] Wang Y, Berardi U, Akbari H (2016). Comparing the effects of urban heat island mitigation strategies for Toronto, Canada. Energy Build..

[61] Rasidi MH, Jamirsah N, Said I (2012). Urban green space design affects urban residents' social interaction. Procedia - Social and Behavioral Sciences.

[62] Gao T, Liang H, Chen Y, Qiu L (2019). Comparisons of landscape preferences through three different perceptual approaches. Int. J. Environ. Res. Public Health.

[63] Cortignani R, Gobattoni F, Pelorosso R, Ripa MN (2018). Green payment and perceived rural landscape quality: A cost-benefit analysis in central Italy. Sustainability.

